# Transcriptome Profiling of Petal Abscission Zone and Functional Analysis of an Aux/IAA Family Gene *RhIAA16* Involved in Petal Shedding in Rose

**DOI:** 10.3389/fpls.2016.01375

**Published:** 2016-09-15

**Authors:** Yuerong Gao, Chun Liu, Xiaodong Li, Haiqian Xu, Yue Liang, Nan Ma, Zhangjun Fei, Junping Gao, Cai-Zhong Jiang, Chao Ma

**Affiliations:** ^1^Beijing Key Laboratory of Development and Quality Control of Ornamental Crops, Department of Ornamental Horticulture, China Agricultural UniversityBeijing, China; ^2^Robert W. Holley Center for Agriculture and Health, United States Department of Agriculture–Agricultural Research ServiceIthaca, NY, USA; ^3^Boyce Thompson InstituteIthaca, NY, USA; ^4^Crops Pathology and Genetic Research Unit, United States Department of Agriculture, Agricultural Research ServiceDavis, CA, USA; ^5^Department of Plant Sciences, University of California at DavisDavis, CA, USA

**Keywords:** *Rosa chinensis*, petal abscission, transcriptome, auxin signaling, *RhIAA16*

## Abstract

Roses are one of the most important cut flowers among ornamental plants. Rose flower longevity is largely dependent on the timing of petal shedding occurrence. To understand the molecular mechanism underlying petal abscission in rose, we performed transcriptome profiling of the petal abscission zone during petal shedding using Illumina technology. We identified a total of 2592 differentially transcribed genes (DTGs) during rose petal shedding. Gene ontology term enrichment and pathway analysis revealed that major biochemical pathways the DTGs were involved in included ethylene biosynthesis, starch degradation, superpathway of cytosolic glycolysis, pyruvate dehydrogenase and TCA cycle, photorespiration and the lactose degradation III pathway. This suggests that alterations in carbon metabolism are an important part of rose petal abscission. Among these DTGs, approximately 150 genes putatively encoding transcription factors were identified in rose abscission zone. These included zinc finger, WRKY, ERF, and Aux/IAA gene families, suggesting that petal abscission involves complex transcriptional reprogramming. Approximately 108 DTGs were related to hormone pathways, of which auxin and ethylene related DTGs were the largest groups including 52 and 41 genes, respectively. These also included 12 DTGs related to gibberellin and 6 DTGs in jasmonic acid pathway. Surprisingly, no DTGs involved in the biosynthesis/signaling of abscisic acid, cytokinin, brassinosteroid, and salicylic acid pathways were detected. Moreover, among DTGs related to auxin, we identified an Aux/IAA gene *RhIAA16* that was up-regulated in response to petal shedding. Down-regulation of *RhIAA16* by virus-induced gene silencing in rose promoted petal abscission, suggesting that *RhIAA16* plays an important role in rose petal abscission.

## Introduction

Plant organ abscission is a crucial process that occurs throughout the life span of plants, and regulates the detachment of organs from main body (Roberts et al., 2002). This will benefit plants for recycling nutrients for continuous growth, propagating, facilitating reproduction, and preventing from disease infections (Addicott, 1982; Nakano et al., 2013). In particular, flower, fruit, and seed abscission is closely correlated with plant reproductive success, crop quality, and productivity (Addicott, 1982; Roberts et al., 2002; Estornell et al., 2013; Nakano and Ito, 2013).

Initiation of flower organ abscission is triggered by both internal and external cues (Taylor and Whitelaw, 2001). As internal cues, interaction of auxin and ethylene plays a critical role in abscission initiation (Meir et al., 2010). Depletion of the polar flow of auxin passing through the abscission zone (AZ) makes the AZ sensitive to ethylene, which triggers the separation process (Abeles and Rubinstein, 1964; Addicott, 1982; Taylor and Whitelaw, 2001). Ethylene biosynthesis and signal transduction pathways are involved in the regulation of abscission. In *Arabidopsis*, ethylene-insensitive mutants *etr1-1*and *ein2* inhibited flower organ shedding, suggesting the roles of ETR1 and EIN2 in abscission (Patterson and Bleecker, 2004). In tomato, repression of EIN3-like gene *LeEIL* expression retarded the flower pedicel abscission and fruit ripening processes (Tieman et al., 2001). Tomato *never ripe (nr)*, *sletr1-1*, and *sletr1-2* mutants affected ethylene receptor function and ethylene sensitivity, thereby delayed fruit ripening and organ abscission (Lanahan et al., 1994; Okabe et al., 2011). On the contrary, auxin as a negative regulator of abscission inhibits the cell separation process (Addicott, 1982; Estornell et al., 2013). The change in auxin flow results in the changes of transcript abundance of many genes involved in auxin biosynthesis, signal transduction, and transport. Functional studies of auxin response factors (ARFs) 1, 2, 7, and 19 demonstrated that these transcriptional regulators have functions in floral organ abscission (Ellis et al., 2005; Okushima et al., 2005). However, the roles of other gene families in the auxin pathway in the regulation of the petal abscission process are still largely unknown.

The perception and transduction of auxin signaling involve the cooperative action of several components. Among them, auxin/indole-3-acetic acid (Aux/IAA) proteins act as transcription repressors by dimerizing with auxin response factors (ARFs; Leyser, 2002; Woodward and Bartel, 2005). In presence of auxin, Aux/IAA proteins binding to the transport inhibitor response one/auxin signaling F-box (TIR1/AFB) cause degradation of Aux/IAA proteins, which then releases ARFs to trigger the expression of auxin responsive genes (Kepinski and Leyser, 2002; Woodward and Bartel, 2005). In *Arabidopsis*, analyses of *Aux/IAA* gain-of-function mutants revealed functional redundancy among Aux/IAA members (Overvoorde et al., 2005). However, distinctive expression patterns in different organs and tissues among *Aux/IAA* genes are displayed in several plant species such as rice (Jain et al., 2006), tomato (Audran-Delalande et al., 2012), *Populus* (Kalluri et al., 2007). In addition, functional analyses of *Aux/IAA* homologues in different plant species demonstrated the distinct and diverse roles of Aux/IAA proteins in plant development and growth (Audran-Delalande et al., 2012). In tomato, expression of an Aux/IAA family gene *Sl-IAA3* is auxin- and ethylene-dependent. The phenotypes resulting from *Sl-IAA3*-silenced transgenic tomato suggested that Sl-IAA3 plays a role in interaction of auxin and ethylene in differential growth (Chaabouni et al., 2009a,b). However, the roles of Aux/IAAs in flower petal abscission are not well documented.

Roses are one of the most important cut flowers among ornamental plants. The opening and longevity of rose flower are major factors in determining the ornamental value of rose flower. Moreover, rose flower longevity is largely dependent on the timing of petal shedding occurrence. However, information on the molecular mechanism governing the rose petal abscission is scarce. To date, the regulatory genes in abscission signaling pathway, including *IDA* (Cho et al., 2008), *NEVERSHED* (Liljegren et al., 2009), *EVERSHED* (Leslie et al., 2010), were identified and characterized in model plants by genetic mapping of mutants. This forward genetic technique is difficult and time-consuming to identify and characterize the genes in non-model plant systems including rose. Next generation sequencing technology has become an effective method to investigate the regulatory network of abscission. Transcriptome studies of the flower abscission process were previously performed in several plant species including tomato (Meir et al., 2010; Nakano et al., 2013; Wang et al., 2013; Ma et al., 2015a; Sundaresan et al., 2016), olive (Gil-Amado and Gomez-Jimenez, 2013), melon (Corbacho et al., 2013), apple (Botton et al., 2011; Zhu et al., 2011), and litchi (Li et al., 2013). Most of these studies have focused on dissecting the regulatory mechanism of pedicel abscission that is the last phase of fruit development and ripening (Giovannoni, 2004). Combined with reverse genetic techniques, roles of several genes obtained from those transcriptome data related to abscission have been confirmed, including *SlERF52* (Nakano et al., 2012, 2014) and *KD1* (Ma et al., 2015a). In *Arabidopsis*, HAESA (HAE) and HAESA-LIKE2 (HSL2)-dependent pathways were revealed to be involved in petal AZ by the comparison of the transcriptomes of wild-type and *hae hsl2* double mutant (Niederhuth et al., 2013). However, the mechanism regulating petal abscission in non-model plants is still not well understood.

Here we investigated the transcriptome dynamics of the petal AZ during petal shedding in rose by Illumina technology and dissected the transcriptional network governing the abscission process. Furthermore, we identified and characterized an *Aux/IAA* gene, *RhIAA16*, which we revealed to have an important role in controlling the timing of petal abscission in rose.

## Materials and Methods

### Plant Materials

**FIGURE 1:**
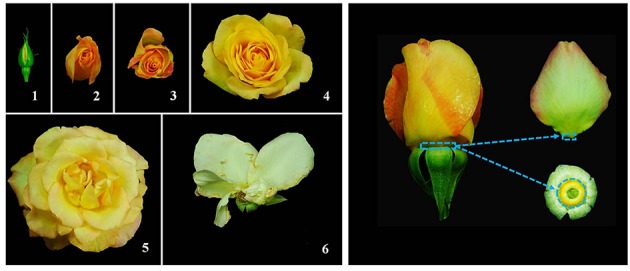
**Flower opening stages in rose (Left), and petal abscission zone region used for RNA-Seq (Right). (Left)** flower opening of rose was divided into six stages: stage 1, partially opened bud; stage 2, completely opened bud; stages 3 and 4, partially opened flower; stage 5, fully opened flower with visible anthers; stage 6, fully opened flower with abscised petals. **(Right)** abscission zone (AZ) sample used for RNA-Seq was the AZ at the base of petal (less than 1 mm in length) and AZ at the receptacle where petals locate (less than 1 mm in length).

Rose flowers (*Rosa chinensis* Jacq. cv. Gold Medal) were grown at a greenhouse on the campus of China Agricultural University, Beijing, China. Rose flower opening was divided into six stages: stage 1, partially opened bud; stage 2, completely opened bud; stages 3 and 4, partially opened flower; stage 5, fully opened flower with visible anthers; stage 6, fully opened flower with abscised petals (**Figure 1**). Rose flowers at different opening stages were harvested. The flower stems were placed immediately in water, and transported to the laboratory within 15 min. The flower stems were re-cut to 20 cm in length under water and placed in deionized water until further processing. The petal was shed at separation layer of abscission zone. Both distal and proximal sides of separation layer belong to abscission zone. Distal side attaches to petal organ, and proximal side attaches to receptacle (**Figure 1**). Therefore, we took sample of petal abscission zone by excising the base of petal (less than 1 mm in length) and the receptacle where petals locate (less than 1 mm in length). Since petals at stage 6 started to abscise, we only focused on the stages prior to petal shedding. Therefore, AZ samples at stages 1, 3, and 5 were collected and used for the transcriptome profiling. Three biological samples were collected for each stage.

Our preliminary tests demonstrated that *R. hybrida* cv. Samantha showed better responses to virus-induced gene silencing (VIGS) and much higher silencing efficiency than *R. chinensis* Jacq. cv. Gold Medal that was used for the transcriptome profiling (data not shown). In addition, the plantlets of *R. hybrida* cv. Samantha bloom as quickly as 40 days after rooting under our growth conditions. Therefore, rose plantlets of *R. hybrida* cv. Samantha were selected for VIGS. Rose plantlets were propagated by tissue culture. Rose shoots with at least 1 node and approximately 2 cm in length were used as explants and cultured on Murashige and Skoog (MS) medium supplemented with 1.0 mg/L 6-Benzyl aminopurine, 3 mg/L Gibberellic Acid, and 0.05 mg/L α-Naphthalene acetic acid for 30 days, then transferred to 1/2-strength MS medium supplemented with 0.1 mg/L NAA for 30 days for rooting.

### Total RNA Extraction and RNA-Seq Library Preparation

Total RNA was extracted using the hot borate method according to previously described (Wan and Wilkins, 1994), and treated with RNase-free DNase I (Promega) to remove any contaminating genomic DNA. Three biological replicates were performed for each developmental stage (stages 1, 3, and 5). Strand-specific RNA libraries were constructed using the protocol described previously (Zhong et al., 2011), and sequenced on a HiSeq 2500 system according to the manufacturer’s instructions. The raw reads were deposited into NCBI SRA database under accession no. PRJNA325324.

### RNA-Seq Data Processing, De novo Assembly and Annotation

RNA-Seq reads were first processed to remove low quality and adaptor sequences using Trimmomatic (Bolger et al., 2014). Reads shorter than 40 bp were removed. The resulting RNA-Seq reads were then aligned to the ribosomal RNA (rRNA) database (Quast et al., 2013) using Bowtie with default parameters (Li and Durbin, 2010). Reads mapped to the rRNA database were discarded. The high-quality cleaned reads were assembled *de novo* into contigs using the Trinity program (Grabherr et al., 2011). To remove the redundancy of Trinity-assembled contigs, the contigs were further assembled *de novo* using iAssembler (Zheng et al., 2011). The final assembled rose contigs were blasted against the UniProt (Swiss-Prot and TrEMBL; The UniProt Consortium, 2014) and *Arabidopsis* protein (version TAIR10) databases (Lamesch et al., 2012) with a cutoff e-value of 1e^-5^. Based on the UniProt and *Arabidopsis* protein blast results, functional descriptions (human readable descriptions) were assigned to each unigene using AHRD^1^. Gene ontology (GO) terms were assigned to the rose assembled transcripts based on the GO terms annotated to their corresponding homologues in the UniProt database. Biochemical pathways were predicted from the rose transcripts using the Pathway Tools (Karp et al., 2002).

To identify differentially expressed genes, high-quality cleaned reads were aligned to rose contigs using Bowtie allowing up to two mismatches. Only the best alignments for each read were retained. Following alignments, raw read count for each rose contig in each sample was derived and normalized to reads per kilo base exon model per million mapped reads (RPKM). The significance of differential gene expression between different samples was determined using DESeq (Anders and Huber, 2010), and raw *p*-values of multiple tests were corrected using false discovery rate (Benjamini and Hochberg, 1995).

### Quantitative RT-PCR

To analyze the transcript abundance of selected genes for confirmation of RNA-Seq data, quantitative RT-PCR (qRT-PCR) reactions were performed, as previously described (Ma et al., 2015b). Briefly, total RNAs were isolated from AZ samples with three biological repeats. 1 μl of the first strand cDNA was used as template with the Step One PlusTM real-time PCR system (Applied Biosystems) using KAPA^TM^ SYBR^®^ FAST quantitative PCR kits (Kapa Biosystems). *RhActin5* was used as a reference gene (Pei et al., 2013). The primers used for determining transcript abundance were listed in **Supplementary Table S1**.

### Virus-Induced Gene Silencing

A 290 bp fragment in the 3′end of *RhIAA16* gene was amplified by PCR from rose cDNAs to specifically silence *RhIAA16*. The fragment was then inserted into pTRV2 vector to generate the pTRV2-*RhIAA16* construct. The primers used for amplifying *RhIAA16* are listed in **Supplementary Table S1**.

Constructs of pTRV1, pTRV2, and pTRV2-*RhIAA16* were transformed into *Agrobacterium tumefaciens* GV3101. *A. tumefaciens* were cultured in Luria-Bertani medium supplemented with 10 mM MES, 20 μM acetosyringone, 50 μg/ml gentamicin sulfate, and 50 μg/ml kanamycin. The cultured bacterium was collected by centrifuge at 4,000 rpm for 10 min, and re-suspended in infiltration buffer (10 mM MgCl_2_, 200 μM acetosyringone, 10 mM MES, pH 5.6) to OD_600_ of ~1.5 (Yin et al., 2015). Mixtures of cultures containing an equal ratio (v/v) of pTRV1 and pTRV2 or pTRV2-*RhIAA16* were used for inoculation. VIGS of rose plantlets was performed as previously described (Tian et al., 2014). Rose plantlets, as shown in **Supplementary Figure S1**, were immersed in bacterial suspension solution and infiltrated under a vacuum at 0.7 MPa for 2 min. After infiltration, plantlets were washed in deionized water, and transplanted into pots containing a mixture of 1:1 (v/v) of peat and vermiculite. The plantlets were immediately placed in dark at 8°C for 3 days in a low temperature incubator (MIR-253, SANYO), and then grown in a culture room at 22 ± 1°C, 40% relative humidity. Three independent experiments were performed. 30 plantlets were used for each experiment. Prior to the petal abscission test, we PCR-screened the plants and determined the transcript abundance of *RhIAA16* in petals among the 30 plantlets. We found that the transcript levels of *RhIAA16* in more than 30% plantlets were reduced, compared to un-inoculated and empty vector controls. These plants with down-regulated *RhIAA16* expression were used for petal abscission assay.

## Results

### Sequencing and *De novo* Assembly of Petal Abscission Zone Transcriptome in Rose

To perform transcriptomic analysis, petal AZs of rose flowers (**Figure 1**) with three biological replicates at stage 1, 3, and 5 were collected to construct a total of nine RNA-Seq libraries. A total of 75,752,884 paired-end raw reads with length of 100 nucleotides (nt) were obtained. After further filtering and cleaning, a total of 57,312,389 clean read pairs were obtained. *De novo* assembly of these high-quality cleaned reads generated 80,226 unique transcripts with an average length of 743 bp (**Table 1**). The size distribution indicated that the lengths of the 19,414 transcripts were more than 1000 bp (**Supplementary Figure S2**). Correlation coefficients of transcriptome profiles among the nine libraries and between the biological replicates were calculated (**Supplementary Table S2**). High correlation coefficients were obtained, suggesting the robustness of our RNA-Seq dataset.

**Table 1:** Summary of rose petal abscission zone transcriptome sequencing dataset.

Items	Total
No. of reads	75,752,884
No. of cleaned reads	57,312,389
No. of mapped reads	44,769,490
No. of assembled transcripts	80,226
Average length of transcripts	743.1 bp
Total length of transcripts	59,617,563 bp

**FIGURE 2:**
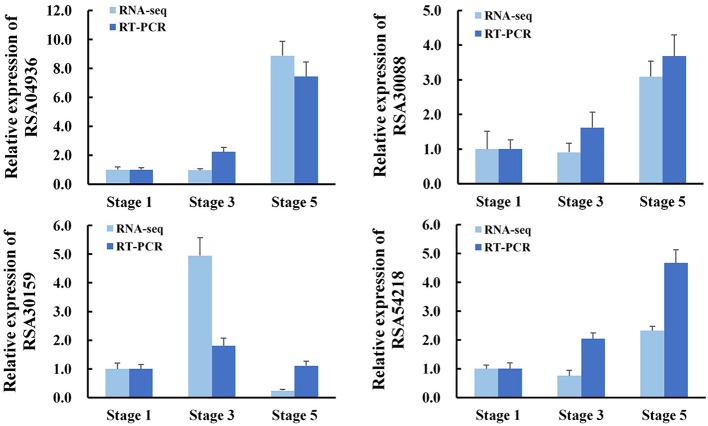
**Validation of RNA-Seq results by quantitative RT-PCR (qRT-PCR).** RNA was extracted from petal AZ at the indicated flower opening stage. *RhActin5* was used as an internal control. The results were the means of three biological replicates ± SD. RSA04936, *GDSL esterase/lipase*; RSA30088, *Receptor-like protein kinase*; RSA30159, *Lachrymatory-factor synthase*; RSA54218, *Zinc transporter*.

To further validate the expression profiles of RNA-Seq data, four selected transcripts were analyzed by qRT-PCR. The results from qRT-PCR analysis were generally in agreement with the expression profiles obtained by RNA-Seq data (**Figure 2**).

### Dynamic Transcriptome Profiles during Petal Abscission in Rose

**FIGURE 3:**
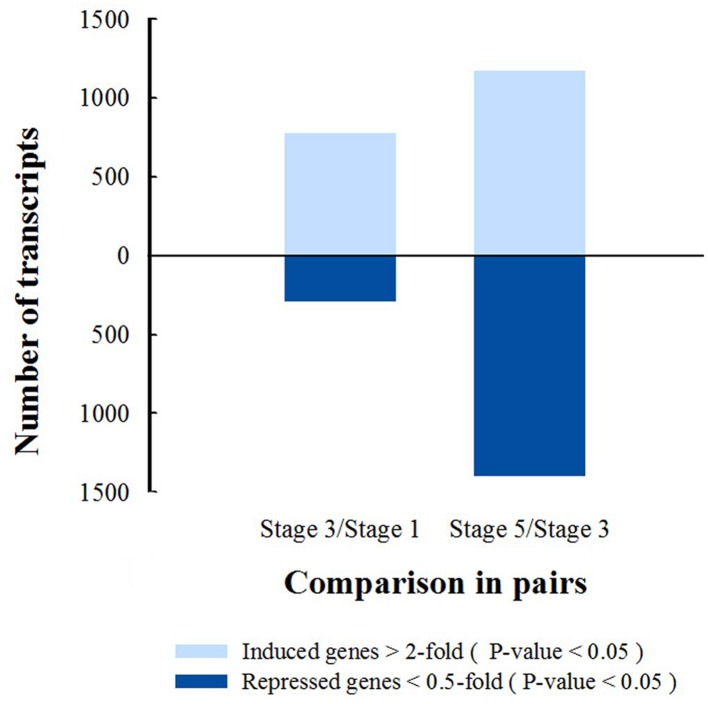
**Numbers of differentially transcribed gene in petal AZ are obtained from comparison of stages 3 and 1, or stages 5 and 3, *P*-value <0.05**.

Differentially transcribed genes (DTGs) were determined using a cutoff ratio of >2 or <0.5 when comparing their transcript abundance in stage 3 to that in stage 1 (S3 vs. S1), and/or in stage 5 to that in stage 3 (S5 vs. S3). A total of 2592 DTGs were obtained (**Supplementary Table S3**). Based on the change in ratio of DTG transcript abundance, the number of DTGs at stage 3/1 and stage 5/3 was counted (**Figure 3**). Compared with stage 1, 782 DTGs were up-regulated and 300 DTGs were down-regulated in stage 3. Compared with stage 3, 1179 transcripts were increased and 1408 transcripts were decreased in stage 5 (**Figure 3**), suggesting that major transcriptional dynamic for petal abscission occurs just prior to petal shedding (stage 5).

**FIGURE 4:**
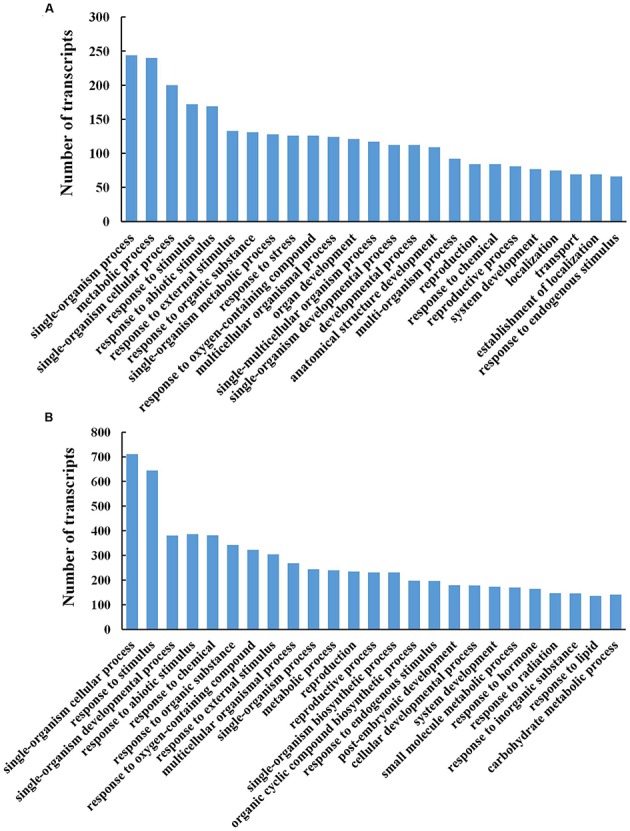
**Gene ontology functional classification analysis of differentially transcribed genes (DTGs) during petal abscission**. Histograms representing functional distributions (GO biological process class) of DTGs obtained from stage 3 compared to stage 1 **(A)**, and from stage 5 compared to stage 3 **(B)**. DTGs were determined using a cutoff ratio of >2 or <0.5 when comparing its expression in stage 3 to that in stage 1 (S3 vs. S1), and/or in stage 5 to that in stage 3 (S5 vs. S3).

To evaluate the potential functions of genes that showed transcriptional changes during petal abscission, we identified the GO terms of the DTGs in the biological process category (**Figure 4**). At stages 3 and 5, the biological processes were enriched in the metabolic process and defense responses including response to abiotic stimulus, external stimulus, organic substance (**Figures 4A,B**).

To identify the biochemical pathways involved in petal abscission, we analyzed DTGs using the Pathway Tools (Karp et al., 2002). The 108 DTGs at stage 3 and 261 DTGs at stage 5 were classified into 42 and 92 biochemical pathways, respectively (**Supplementary Table S4**). The major pathways both at stage 3 and 5 included ethylene biosynthesis, starch degradation, superpathway of cytosolic glycolysis, pyruvate dehydrogenase and TCA cycle, and photorespiration (**Supplementary Table S4**). In addition, one of the major pathways at stage 5 is related to the lactose degradation III pathway (**Supplementary Table S4**). These results suggested that alterations in carbon metabolism play an important part in rose petal abscission.

### Abscission-Responsive Transcriptional Regulators in Rose Petal Abscission Zone

**FIGURE 5:**
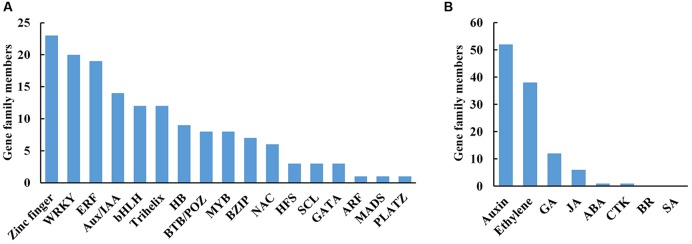
**Distribution of transcription factors (A) and hormone (B) related DTGs during petal abscission.** DTGs were determined using a cutoff ratio of >2 or <0.5 when comparing its expression in stage 3 to that in stage 1 (S3 vs. S1), and/or in stage 5 to that in stage 3 (S5 vs. S3). GA, gibberellin; JA, jasmonate; ABA, abscisic acid; CTK, cytokinin; BR, brassinosteroid; SA, salicylic acid.

Of 2592 DTGs, 150 encoded putative transcription factors (TFs; **Figure 5A**). More specifically, 15.3% (23/150) belonged to zinc finger family, 13.3% (20/150) to WRKYs, 12.7% (19/150) to ERFs (ethylene responsive factors), and 9.3% (14/150) to Aux/IAAs (**Figure 5A**). As the largest group of abscission-responsive TFs, the transcript abundance of most of the zinc finger family members (18/23) was increased in stage 3 or stage 5 (**Supplementary Table S5**). Furthermore, the transcript abundance of all the WRKY family members was induced during rose flower opening (**Supplementary Table S5**). The results suggested a complex transcriptional reprogramming of petal abscission.

### Abscission-Induced Hormone Pathway Changes in Rose Petal Abscission Zone

Hormones act as internal cues to initiate abscission process (Addicott, 1982; Estornell et al., 2013). We identified 108 DTGs related to hormone pathways. Among them, DTGs related to auxin and ethylene pathways including 52 DTGs and 38 DTGs, respectively, were the largest group (**Figure 5B**; **Table 2**; **Supplementary Table S6**). In addition, 12 DTGs related to gibberellin were obtained, and 6 DTGs in jasmonic acid pathway (**Figure 5B**; **Supplementary Table S6**). No DTGs involved in the biosynthesis/signaling of abscisic acid, cytokinin, brassinosteroid, salicylic acid pathways were detected (**Figure 5B**).

**Table 2:** Expression changes of transcripts related auxin pathway in response to abscission.

GeneID	Annotation	Transcript abundance (RPKM)			Ratio Stage 3/1	Ratio Stage 5/3
		Stage 1	Stage 3	Stage 5		
**Auxin transporter**						
RSA03469	Auxin efflux carrier family protein	17.66	32.95	76.18	1.87	2.31
RSA57045	Auxin transporter-like protein	11.79	15.09	33.64	1.28	2.23
RSA40292	Auxin efflux carrier family protein	23.29	32.14	69.9	1.38	2.17
RSA48611	Auxin efflux carrier	106.54	56.24	26.35	0.53	0.47
RSA02921	Auxin efflux carrier family protein	30.53	13.13	2.53	0.43	0.19
RSA22467	Auxin efflux carrier family protein	25.07	10.37	0.27	0.41	0.03
**Aux/IAA family**						
RSA45030	Aux/IAA27-like	42.87	20.59	81.81	0.48	3.97
RSA47413	Aux/IAA26-like	8.76	5.37	19.73	0.61	3.67
RSA38191	Aux/IAA27-like	58.7	28.52	101.47	0.49	3.56
RSA04500	Aux/IAA27	33.66	33.19	88.58	0.99	2.67
RSA33069	Aux/IAA16	73.94	80.94	165.03	1.09	2.04
RSA52969	Aux/IAA8-like	55.95	62.56	27.42	1.12	0.44
RSA45028	Aux/IAA28-like	79.15	394.48	169.38	4.98	0.43
RSA45029	Aux/IAA28-like	85.33	416.25	177.08	4.88	0.43
RSA45026	Aux/IAA28-like	78.92	394.68	168.36	5	0.43
RSA45027	Aux/IAA28-like	83.91	411.14	173.63	4.9	0.42
RSA05184	Aux/IAA1-like	1.79	5.96	0.67	3.32	0.11
RSA37816	Aux/IAA13 isoform X1	33.29	13.14	6.55	0.39	0.5
RSA37817	Aux/IAA13 isoform X1	31.83	13.3	6.68	0.42	0.5
RSA63979	Aux/IAA13 isoform X1	9.72	7.96	16.83	0.82	2.11
**Others**						
RSA29268	Auxin-regulated protein	6.83	4.76	19.07	0.7	4
RSA55555	Auxin-induced in root cultures protein 12-like	18.79	22.87	89.63	1.22	3.92
RSA40153	Auxin-induced protein 5NG4, putative	4.31	3	11.37	0.7	3.79
RSA55554	Auxin-induced in root cultures protein 12-like	13.27	14.59	53.88	1.1	3.69
RSA47053	Auxin response factor three family protein	30.04	14.91	35.9	0.5	2.41
RSA46802	Auxin-responsive protein SAUR36	2.36	11.11	26.6	4.7	2.4
RSA46801	Auxin-responsive protein SAUR36	2.16	7.63	18.02	3.53	2.36
RSA01454	Auxin-induced protein 5NG4, putative	25.62	11.88	3.61	0.46	0.3
RSA01453	Auxin-induced protein 5NG4, putative	28.64	14.67	3.93	0.51	0.27
RSA64224	Auxin-induced protein 15A-like	4.11	9.56	2.56	2.32	0.27
RSA64234	Auxin-induced protein-like protein	9.62	13.79	3.57	1.43	0.26
RSA06545	Auxin-induced protein-like protein	4.69	6.24	1.5	1.33	0.24
RSA64231	Auxin-induced protein 15A-like	8.15	12.1	2.9	1.48	0.24
RSA37530	Auxin-induced protein 15A-like	10.56	7.87	1.72	0.74	0.22
RSA64235	Auxin-induced protein 15A-like	7.93	8.89	1.87	1.12	0.21
RSA64222	Auxin-induced protein 15A-like	5.06	9.98	1.78	1.97	0.18
RSA58610	Auxin-induced protein 22D-like	1.13	37.04	6.43	32.69	0.17
RSA06546	Auxin-induced protein 15A-like	4.57	8.65	1.36	1.89	0.16
RSA64232	Auxin-induced protein 15A-like	5.75	9.72	1.42	1.69	0.15
RSA64228	Auxin-induced protein 15A-like	5.98	10.76	1.66	1.8	0.15
RSA10174	Auxin-responsive protein	2.33	7.69	1.17	3.3	0.15
RSA64229	Auxin-induced protein 15A-like	4.31	6.99	0.92	1.62	0.13
RSA64230	Auxin-induced protein 15A-like	5.42	8.03	0.87	1.48	0.11
RSA64233	Auxin-induced protein 15A-like	6.44	10.1	1.15	1.57	0.11
RSA64225	Auxin-induced protein 15A-like	6.7	9.89	1.11	1.48	0.11
RSA54814	Auxin-induced protein 15A-like	3.75	6.04	0.69	1.61	0.11
RSA54813	Auxin-induced protein 15A-like	3.5	5.26	0.54	1.51	0.1
RSA64226	Auxin-induced protein 15A-like	5.57	8.26	0.86	1.48	0.1
RSA54803	Auxin-induced protein 15A-like	2.74	6.44	0.55	2.35	0.09
RSA54811	Auxin-induced protein ARG7-like	1.87	5.13	0.46	2.74	0.09
RSA39307	Early auxin response protein	2.17	12.55	1.19	5.79	0.09
RSA31518	Auxin-responsive protein SAUR71-like	1.09	183.12	0.85	168.52	0

Among DTGs related to auxin pathway, six auxin transporter genes were identified including five auxin efflux carrier genes (**Table 2**). In addition, 14 Aux/IAA family members were obtained, of which six members were up-regulated in stage 5, and eight members were down-regulated in stage 5 compared to stage 3 (**Table 2**). Among DTGs related to ethylene pathway, 15 DTGs encoded ethylene biosynthesis related 1-Aminocyclopropane-1-carboxylic acid oxidase (ACO). Transcript abundance of 10 *ACO* genes was accumulated in stage 5 compared to stage 3. Furthermore, 19 DTGs encoded ERFs were detected (**Supplementary Table S6**). The transcript abundance of 16 *ERFs* was increased in stage 5 compared to stage 3 (**Supplementary Table S6**). Overall, our results suggested that auxin and ethylene may play central roles in petal abscission of rose.

### *RhIAA16* Transcript Abundance Is Induced during Petal Abscission

**FIGURE 6:**
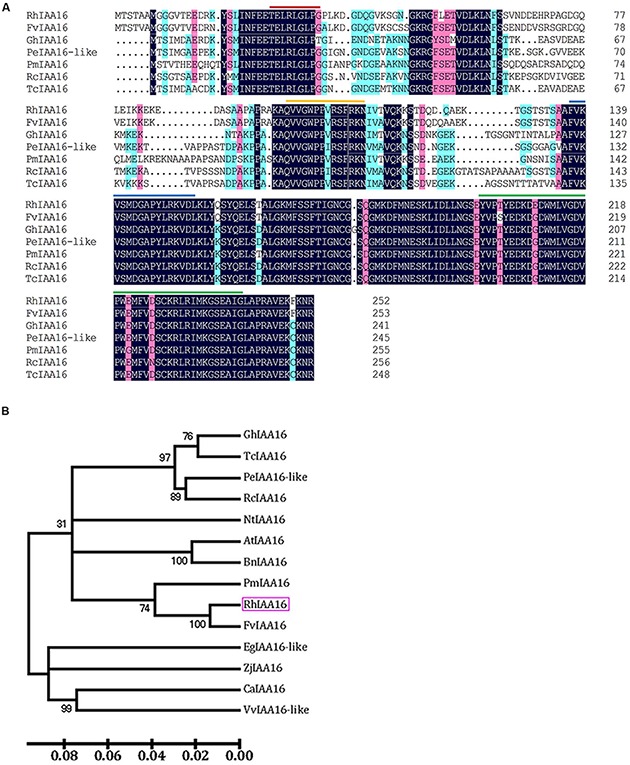
**Alignment of deduced amino acid sequences (A) and phylogenetic tree (B) of RhIAA16 protein and representative Aux/IAA members.** Lines indicated representatively conserved motif, including red line, domain I; yellow line, domain II; blue line, domain III; green line, domain IV (Audran-Delalande et al., 2012).

**FIGURE 7:**
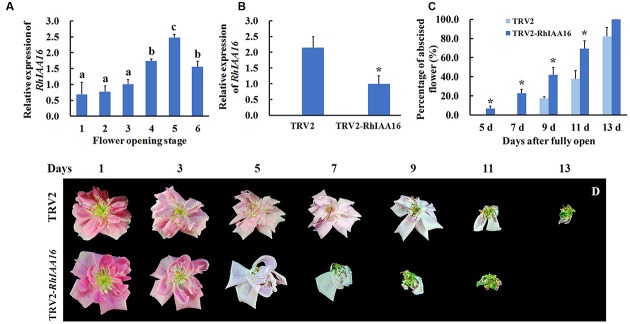
**Silencing of *RhIAA16* promotes petal abscission. (A)** Expression of *RhIAA16* during flower opening was analyzed by qRT-PCR. **(B)** Expression of *RhIAA16* was analyzed by qRT-PCR in *RhIAA16*-silenced (TRV2-RhIAA16) and control (TRV2) plants. **(C)** The percentage of abscised flowers were determined at intervals after fully open in *RhIAA16*-silenced (TRV2-RhIAA16) and control (TRV2) plants. Abscised flower was defined as the flower with all the petals shed. **(D)** The phenotypes of flowers were recorded and photographed every 2 days. The results were the means of three biological replicates with standard deviation. Letters indicated significant differences according to Duncan’s multiple range test (*P* < 0.05), and asterisks indicated statistically significant differences (Student’s *t*-test, *P* < 0.05).

To identify key regulators governing petal abscission, we initiated functional analysis of DTGs using VIGS. We primarily focused on the up-regulated DTGs, hypothesizing that VIGS-down regulation of these genes might lead to changes in the petal abscission processes. Given the potential important roles that auxin plays in the regulation of abscission, we first examined the functions of several Aux/IAA genes (RSA33069, RSA04500, RSA45030) that were up-regulated in the rose abscission zone using rose cut flowers. We found that VIGS-silencing of the contig of RSA33069 exhibited accelerated petal abscission phenotype. Analysis of the RSA33069 cDNA sequence revealed that it encoded a deduced protein of 253 amino acids with a 762 bp predicted open reading frame (**Figure 6A**). The predicted amino acid sequence of RSA33069 showed that it belongs to the Aux/IAA family, and has the four canonical conserved domains known for this family (**Figure 6A**) (Overvoorde et al., 2005). In addition, phylogenetic tree analysis suggested that the protein has high degree of sequence homology to FvIAA16 from *Fragaria vesca*, therefore was designated as RhIAA16 (**Figure 6B**). RT-PCR analysis demonstrated that the transcript abundance of *RhIAA16* in petal AZ was significantly induced during flower development to peak at stage 5 (**Figure 7A**). These results were consistent with the transcript abundance changes of *RhIAA16* in the RNA-Seq data.

### Reduction of *RhIAA16* Expression Promotes Petal Abscission in Rose

To further confirm the potential role of *RhIAA16* in petal abscission, we chose a fragment from *RhIAA16*-specific 3′ un-translated region (UTR) to silence *RhIAA16* in rose plantlets. qRT-PCR results showed that transcript abundance of *RhIAA16* in *RhIAA16*-silenced (TRV2-RhIAA16) petal was significantly reduced compared to TRV2 control (**Figure 7B**). Petal abscission was detected at 5 days after full opening in the *RhIAA16*-silenced plantlets in contrast to 9 days after full opening in TRV2 control plantlets (**Figures 7C,D**), suggesting that silencing of *RhIAA16* accelerated the timing of initial petal abscission. At 9 days and 11 days after full opening, petals in ~40.5 and 69.0% of flowers in *RhIAA16*-silenced plantlets had abscised whereas petals in only 17.8 and 37.9% of TRV2 control flowers were abscised, respectively (**Figure 7C**).

## Discussion

In this study, high-throughput sequencing and *de novo* assembly strategies permitted us to dissect the transcriptome of rose petal AZ during petal shedding. Our results demonstrated that among DTGs related to hormones during petal abscission, most of them were associated with auxin and ethylene pathways, suggesting that auxin and ethylene play important roles in petal abscission in rose flowers (**Figure 5B**). This conclusion is in good agreement with tomato abscission studies (Meir et al., 2010). Furthermore, functional characterization of *RhIAA16* partially supports this notion (**Figure 7**).

Auxin and ethylene as key hormones in the initiation of abscission have been demonstrated not only by physiological experiments, but also by transcriptome studies from different organ AZ. In tomato pedicel AZ, auxin depletion by auxin transport inhibitor, or flower removal stimulated pedicel abscission while ethylene action inhibitor treatment prevented the abscission induced by auxin depletion (Meir et al., 2010). In addition, the transcriptome of pedicel AZ demonstrated that acquisition of ethylene sensitivity in the AZ is associated with altered expression of auxin-regulated genes (Meir et al., 2010). Auxin homeostasis and signaling are usually modulated by the *Aux/IAA* genes (Song et al., 2009). Canonical Aux/IAA proteins function as transcriptional repressors of auxin-regulated genes (Tiwari et al., 2001, 2004). In our study, the DTGs included 11 Aux/IAA family members (**Table 2**). Among them, the transcript abundance of six members of Aux/IAA family was up-regulated during petal abscission (**Table 2**). Intriguingly, VIGS-silencing *RhIAA16*, one of the up-regulated *Aux/IAA* genes, accelerated the petal abscission process (**Figure 7**), suggesting that RhIAA16 might be required for preventing premature abscission. In soybean, *IAA16* has been reported as abscission-specific transcription factor by a transcriptome analysis of soybean leaf abscission, although the expression of *IAA16* was down-regulated during soybean leaf abscission (Kim et al., 2016). In *Arabidopsis*, genetic evidences suggest that ARFs, which interact with Aux/IAA proteins, play regulatory roles in the petal abscission process. ARF1, ARF2, ARF7, and ARF19 were identified as regulators of abscission (Ellis et al., 2005). Given that ARF proteins interact with Aux/IAA in the auxin signal pathway (Leyser, 2002), further characterization of interactions between RhIAA16 and ARFs in rose may shed light on petal abscission activation.

It is worth pointing out that the transcript levels of *RhIAA16* were not significantly changed in response to ethylene and ethylene action inhibitor 1-Methylcyclopropene (1-MCP) treatments (data not shown), suggesting that *RhIAA16* is involved in either an ethylene independent pathway or up-stream of the ethylene pathway during abscission initiation.

In tomato, microarray assay showed that the expression of genes related to different steps of ethylene biosynthetic pathway, including *S-adenosylmethionine* (*SAM*) *synthase*, *ACC synthase*, and *ACO* genes, were altered during the pedicel abscission process (Meir et al., 2010). However, our transcriptome study revealed that multiple *ACO* genes in the final step of ethylene biosynthesis (Yang and Hoffman, 1984) were increased in AZ of stage 5 flower (**Supplementary Table S6**), indicating the critical role of *ACO* in controlling the rose petal abscission. Furthermore, the transcript abundance of many *ERFs* was induced in stage 5 compared with stage 3 (**Supplementary Table S6**). The importance of ERFs in flower abscission was recently demonstrated in tomato (Nakano et al., 2014). These researchers found that silencing of *SlERF52*, which is specifically expressed in the pedicel AZ, delayed tomato flower pedicel abscission.

In *Arabidopsis*, the transcriptomic analysis of petal abscission indicated that ethylene and ABA pathways were enriched during petal abscission (Niederhuth et al., 2013). In addition, JA signaling and biosynthesis genes were also involved in petal abscission (Niederhuth et al., 2013). Functional analysis showed that mutants of JA biosynthesis gene *allene oxide synthase* (*AOS*) retarded the petal abscission (Kim et al., 2013). This delayed abscission phenotype can be enhanced by ethylene insensitive mutant *ein2* and ABA deficient mutant *aba2*, suggesting that ethylene, ABA and JA might synergistically regulate the petal abscission in *Arabidopsis* (Ogawa et al., 2009; Kim et al., 2013). In our study, the transcriptomic analysis suggested that among these three hormones, ethylene might play a major role, and JA might also be recruited in rose petal abscission, but not ABA (**Figure 5**). In GO analysis of our transcriptome showed that defense responses such as response to abiotic stimulus and external stimulus were enriched during petal abscission process (**Figure 4**), suggesting that the genes responsive to stress are involved in the activation of abscission. Indeed, abscission is considered to be a physiological process related to hormone-mediated stress responses (Addicott, 1982; Estornell et al., 2013). In *Arabidopsis*, GO enrichment analysis of petal AZ transcriptome also demonstrated that biological processes that significantly enriched include defense response to abiotic and biotic stresses (Niederhuth et al., 2013). Similarly, in soybean, the biological processes of leaf AZ transcriptome significantly enriched include responses to endogenous and external stimuli (Kim et al., 2016).

Zinc finger transcription factors are a large and diverse family involved in many aspects of plant growth and development and play critical roles in cellular functions such as transcriptional regulation, RNA binding, and protein-protein interactions (Ciftci-Yilmaz and Mittler, 2008). Our results showed that zinc finger genes are the largest group among differentially transcribed transcription factors in rose petal AZ (**Figure 5A**). In *Arabidopsis*, *ZINC FINGER PROTEIN2* (*ZFP2*) encoding a ZFP, has been revealed in stamen AZ transcriptome profiling, which was increased during floral organ abscission process. Overexpression of *ZFP2* exhibited delayed floral organ abscission phenotype (Cai and Lashbrook, 2008). Our data showed that 18 members of zinc finger gene were elevated in stage 5 (**Supplementary Table S5**), indicated the important roles of zinc finger gene in rose petal abscission. Functional analysis of these regulatory genes would be an important step toward elucidating their roles in petal abscission in the future.

## Conclusion

Our RNA-Seq analysis has permitted us to dissect the rose AZ transcriptome during petal abscission, and reveal that auxin and ethylene are important hormones in the regulation of the abscission process. Furthermore, our data demonstrated that an *Aux/IAA* gene, *RhIAA16*, played an important role in rose petal abscission.

## Author Contributions

CM and C-ZJ conceived and supervised the study. YG, CL, XL, HX, and YL performed the experiments. YG and CL analyzed the data and drafted the manuscript. CM, NM, ZF, C-ZJ, and JG provided technical support, conceptual advice, analyzed the data, and participated in writing the manuscript. C-ZJ extensively revised the manuscript.

## Conflict of Interest Statement

The authors declare that the research was conducted in the absence of any commercial or financial relationships that could be construed as a potential conflict of interest.

## Footnotes

1 https://github.com/asishallab/AHRD-1

## Supplementary material

FIGURE S1 **Rose seedling for virus-induced gene silencing**.

FIGURE S2 **Length distribution of rose petal abscission zone unique transcripts**.

TABLE S1 **The primer list**.

TABLE S2 **Correlation coefficients of transcriptome profiles among RNA-Seq samples**.

TABLE S3 **Differentially transcribed genes**.

TABLE S4 **Pathways of differentially transcribed genes**.

TABLE S5 **Differentially transcribed transcription factor**.

TABLE S6 **Differentially transcribed genes related to hormones**.
