# Supplementary figures and images for: Transcriptome Profiling of Petal Abscission Zone and Functional Analysis of an Aux/IAA Family Gene RhIAA16 Involved in Petal Shedding in Rose
Source: Front Plant Sci. 2016 Sep 15;7:1375. doi: 10.3389/fpls.2016.01375 (PMC5023668; doi:10.3389/fpls.2016.01375)

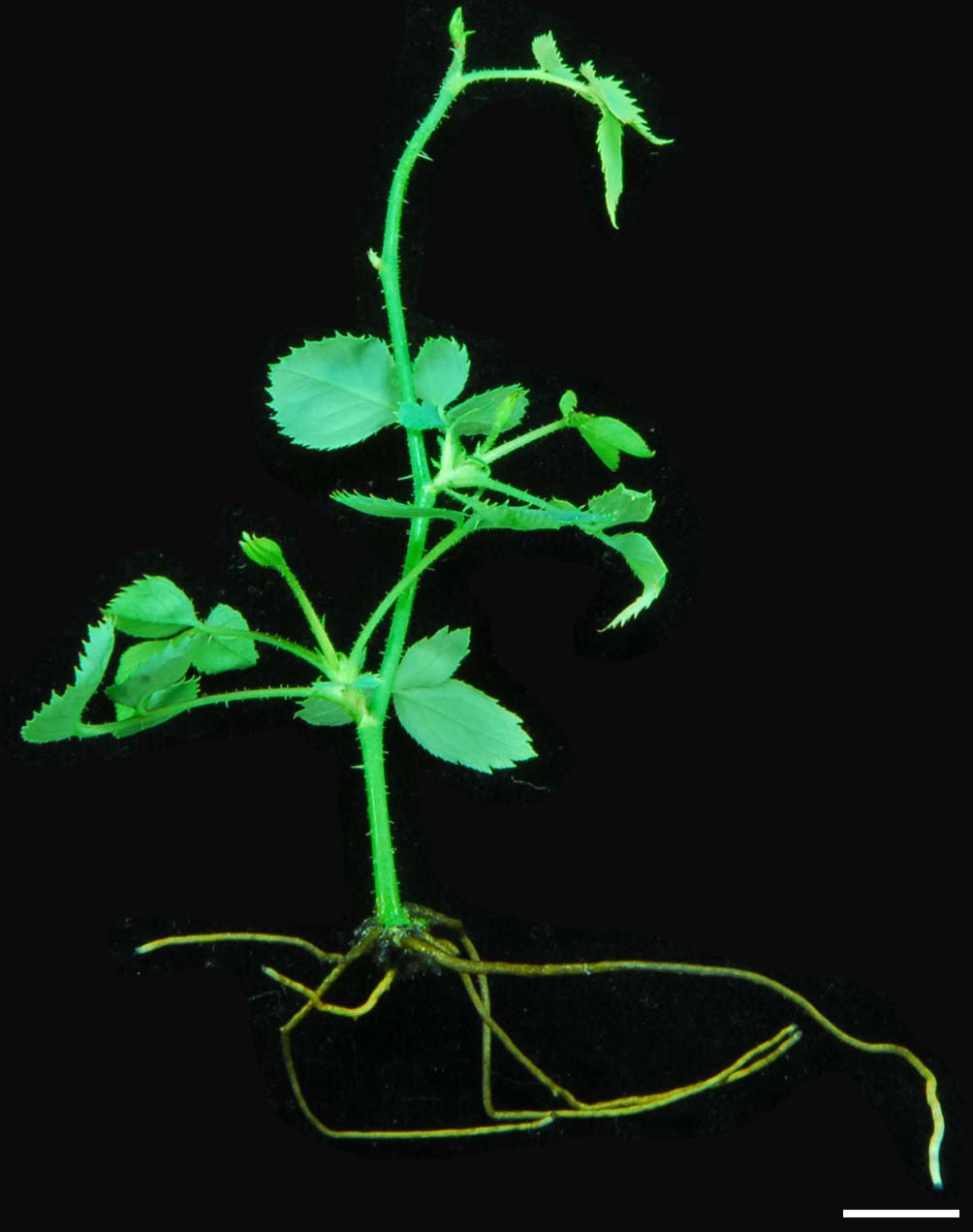

Supplement: FIGURE S1 — Rose seedling for virus-induced gene silencing. [file Image_1.JPEG]

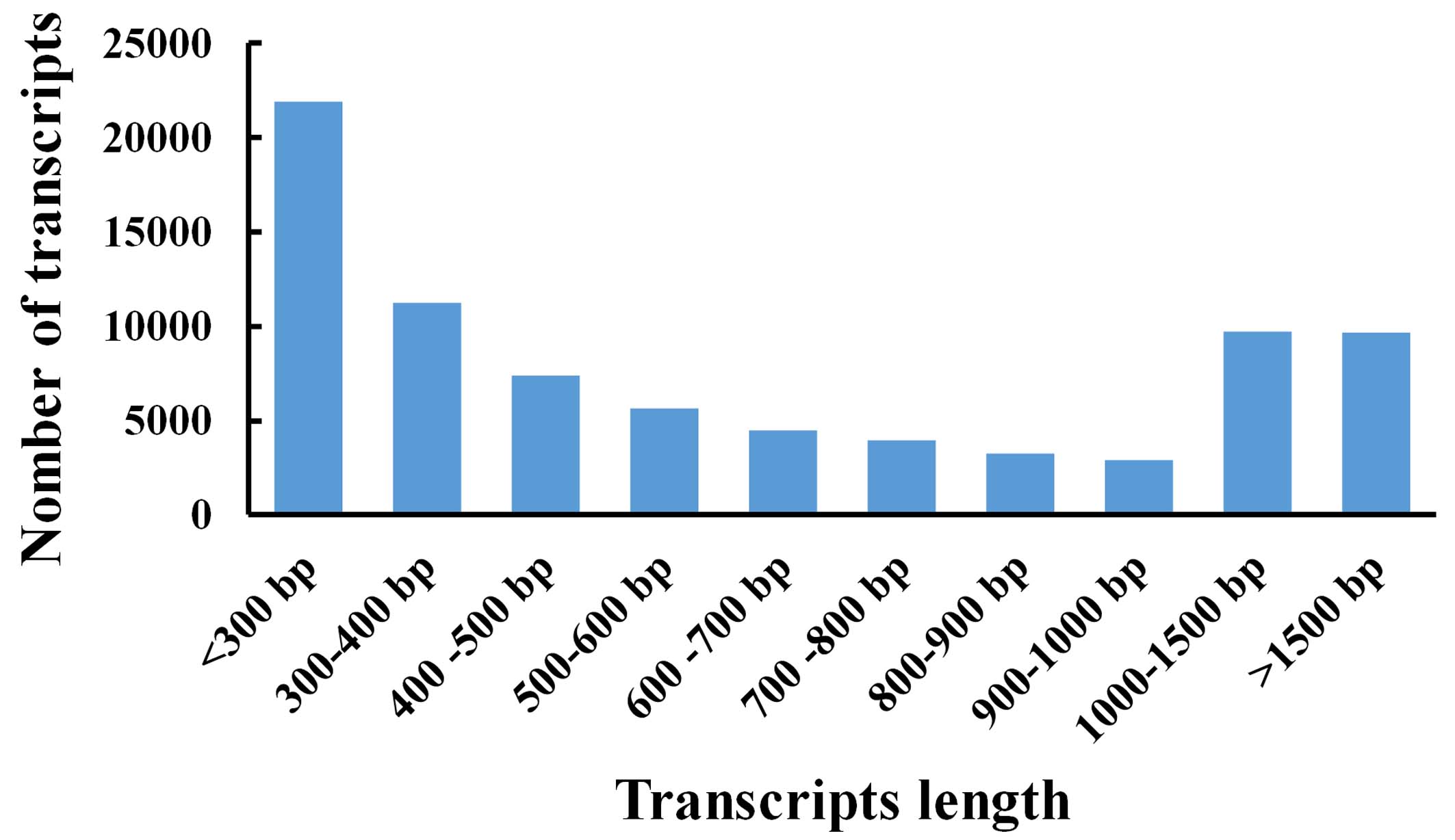

Supplement: FIGURE S2 — Length distribution of rose petal abscission zone unique transcripts. [file Image_2.JPEG]
