# Supplementary material for: Transcriptome Profiling of Petal Abscission Zone and Functional Analysis of an Aux/IAA Family Gene RhIAA16 Involved in Petal Shedding in Rose
Source: Front Plant Sci. 2016 Sep 15;7:1375. doi: 10.3389/fpls.2016.01375 (PMC5023668; doi:10.3389/fpls.2016.01375)
Supplement: TABLE S1 — The primer list. [file Table_1.DOCX]

***Supplementary Material***

**Transcriptome profiling of petal abscission zone and functional analysis of an Aux/IAA family gene *RhIAA16* involved in petal shedding in rose**

**Yuerong Gao, Chun Liu, Xiaodong Li, Haiqian Xu, Yue Liang, Nan Ma, Zhangjun Fei, Junping Gao, Cai-Zhong Jiang, Chao Ma**

***Correspondence:**

Chao Ma ([mac@cau.edu.cn](mailto:mac@cau.edu.cn)) & Cai-Zhong Jiang ([cjiang@ucdavis.edu](mailto:cjiang@ucdavis.edu))

**Supplementary Table 1. The primer list**

| **Accession No. or gene name** | **Primer set** | |
| --- | --- | --- |
|  | **Forward primer (5’-3’)** | **Reverse primer (5’-3’)** |
| **For qRT-PCR verification of database results** | | |
| RSA04936 | GTGGAGCCATACTCCCGATG | GCAGTTATAACACCCGCAGC |
| RSA54218 | GCTACATTGGGGTTCAGTGGA | AAACATGGGGACCAGCCAAA |
| RSA30088 | CCAGTGGTACCCGGTGAATC | GGCATGTAGACAAGCATCAGT |
| RSA30159 | TCAGGCGGTGTTTGAGCTAC | CGGCGGCTTTGGTCGTTAT |
| RSA33069 | CAAAGGCTCAAGTTGTGGGTT | AACCAAGCAGACGAAGCGAG |
| RhActin5 | GAGCGTTTCAGATGCCCAGA | TGGTGGGGCAACCACCTTA |
| **For construction of vector** | | |
| RhIAA16 | GTAGAATTCTGAAGATGAAGCTGCTCGC | TATCTCGAGTTAAACAGGCTGCCGTAGACA |
