# Supplementary material for: Transcriptome Profiling of Petal Abscission Zone and Functional Analysis of an Aux/IAA Family Gene RhIAA16 Involved in Petal Shedding in Rose
Source: Front Plant Sci. 2016 Sep 15;7:1375. doi: 10.3389/fpls.2016.01375 (PMC5023668; doi:10.3389/fpls.2016.01375)
Supplement: TABLE S2 — Correlation coefficients of transcriptome profiles among RNA-Seq samples. [file Table_2.DOCX]

***Supplementary Material***

**Transcriptome profiling of petal abscission zone and functional analysis of an Aux/IAA family gene *RhIAA16* involved in petal shedding in rose**

**Yuerong Gao, Chun Liu, Xiaodong Li, Haiqian Xu, Yue Liang, Nan Ma, Zhangjun Fei, Junping Gao, Cai-Zhong Jiang, Chao Ma**

***Correspondence:**

Chao Ma ([mac@cau.edu.cn](mailto:mac@cau.edu.cn)) & Cai-Zhong Jiang ([cjiang@ucdavis.edu](mailto:cjiang@ucdavis.edu))

**Supplementary Table 2. Correlation coefficients of transcriptome profiles among RNA-Seq samples**

| **Sample** | **GM1_rep1** | **GM1_rep2** | **GM1_rep3** | **GM3_rep1** | **GM3_rep2** | **GM3_rep3** | **GM5_rep1** | **GM5_rep2** | **GM5_rep3** |
| --- | --- | --- | --- | --- | --- | --- | --- | --- | --- |
| **GM1_rep1** | 1 | 0.95 | 0.95 | 0.49 | 0.45 | 0.5 | 0.4 | 0.33 | 0.36 |
| **GM1_rep2** | 0.95 | 1 | 0.97 | 0.61 | 0.54 | 0.59 | 0.47 | 0.41 | 0.43 |
| **GM1_rep3** | 0.95 | 0.97 | 1 | 0.48 | 0.45 | 0.48 | 0.34 | 0.3 | 0.31 |
| **GM3_rep1** | 0.49 | 0.61 | 0.48 | 1 | 0.94 | 0.98 | 0.7 | 0.67 | 0.66 |
| **GM3_rep2** | 0.45 | 0.54 | 0.45 | 0.94 | 1 | 0.96 | 0.53 | 0.49 | 0.47 |
| **GM3_rep3** | 0.5 | 0.59 | 0.48 | 0.98 | 0.96 | 1 | 0.68 | 0.63 | 0.63 |
| **GM5_rep1** | 0.4 | 0.47 | 0.34 | 0.7 | 0.53 | 0.68 | 1 | 0.96 | 0.98 |
| **GM5_rep2** | 0.33 | 0.41 | 0.3 | 0.67 | 0.49 | 0.63 | 0.96 | 1 | 0.98 |
| **GM5_rep3** | 0.36 | 0.43 | 0.31 | 0.66 | 0.47 | 0.63 | 0.98 | 0.98 | 1 |
