# Supplementary material for: Transcriptome Profiling of Petal Abscission Zone and Functional Analysis of an Aux/IAA Family Gene RhIAA16 Involved in Petal Shedding in Rose
Source: Front Plant Sci. 2016 Sep 15;7:1375. doi: 10.3389/fpls.2016.01375 (PMC5023668; doi:10.3389/fpls.2016.01375)
Supplement: TABLE S4 — Pathways of differentially transcribed genes. [file Table_4.DOCX]

***Supplementary Material***

**Transcriptome profiling of petal abscission zone and functional analysis of an Aux/IAA family gene *RhIAA16* involved in petal shedding in rose**

**Yuerong Gao, Chun Liu, Xiaodong Li, Haiqian Xu, Yue Liang, Nan Ma, Zhangjun Fei, Junping Gao, Cai-Zhong Jiang, Chao Ma**

***Correspondence:**

Chao Ma ([mac@cau.edu.cn](mailto:mac@cau.edu.cn)) & Cai-Zhong Jiang ([cjiang@ucdavis.edu](mailto:cjiang@ucdavis.edu))

**Supplementary Table 4 Pathways of differentially transcribed genes**

| **Pathway ID** | **Specific pathway at stage 3** | **No. of genes** |
| --- | --- | --- |
| PWY-181 | Photorespiration | 11 |
| PWY-6737-58 | Starch degradation V | 10 |
| PWY-5464 | Superpathway of cytosolic glycolysis (plants), pyruvate dehydrogenase and TCA cycle | 9 |
| TRIGLSYN-PWY | Triacylglycerol biosynthesis | 8 |
| PWY-5143 | Fatty acid activation | 6 |
| ETHYL-PWY-35 | Ethylene biosynthesis I | 6 |
| PWY-6842-12 | Glutathione-mediated detoxification II | 5 |
| PWY-6352 | 3-phosphoinositide biosynthesis | 4 |
| PWY1F-FLAVSYN-39 | Flavonoid biosynthesis | 3 |
| PWY-6900-10 | (Z)-butanethiol-S-oxide biosynthesis | 3 |
| PWY0-1335-16 | NADH to cytochrome bo oxidase electron transfer NADH | 2 |
| PWY-6475-33 | Trans-lycopene biosynthesis II | 2 |
| PWY-6902-58 | Chitin degradation II | 2 |
| PWY-735-20 | Jasmonic acid biosynthesis | 2 |
| PWY-5349 | Esculetin biosynthesis | 2 |
| BGALACT-PWY-60 | Lactose degradation III | 2 |
| ARO-PWY | Chorismate biosynthesis I | 2 |
| PWY-6443-6 | Benzoate biosynthesis I (CoA-dependent, &beta;-oxidative) | 2 |
| PWY-241 | C4 photosynthetic carbon assimilation cycle, NADP-ME type | 2 |
| PWY-5070-17 | Gibberellin biosynthesis I (non C-3, non C-13 hydroxylation) | 2 |
| PWY-5148 | Acyl-CoA hydrolysis | 2 |
| PWY-6803-25 | Phosphatidylcholine acyl editing | 1 |
| FASYN-ELONG-PWY-26 | Fatty acid elongation -- saturated | 1 |
| PWY-6323-35 | Benzoylanthranilate biosynthesis | 1 |
| PWY-5910-3 | Superpathway of geranylgeranyldiphosphate biosynthesis I (via mevalonate) | 1 |
| PWY-5787-44 | Oligomeric urushiol biosynthesis | 1 |
| ILEUSYN-PWY | Isoleucine biosynthesis I (from threonine) | 1 |
| LIPAS-PWY | Triacylglycerol degradation | 1 |
| PWY-4321-54 | Glutamate degradation IV | 1 |
| PWY-5461-56 | Betanidin degradation | 1 |
| PWY-1081-59 | Homogalacturonan degradation | 1 |
| PWY-801-1 | Homocysteine and cysteine interconversion | 1 |
| POLYAMINSYN3-PWY-2 | Superpathway of polyamine biosynthesis II | 1 |
| PWY-1121 | Suberin monomers biosynthesis | 1 |
| PWY-7214-8 | Baicalein degradation (hydrogen peroxide detoxification) | 1 |
| PWY-6692-14 | Fe(II) oxidation | 1 |
| PWY-5466-24 | Matairesinol biosynthesis | 1 |
| PWY-6515-25 | Phloridzin biosynthesis | 1 |
| PWY0-541-31 | Cyclopropane-fatty-acyl-phospholipid synthase family protein | 1 |
| PWY-7250-38 | [2Fe-2S] iron-sulfur cluster biosynthesis | 1 |
| PWY-7170-39 | Phytochromobilin biosynthesis | 1 |
| PWY-7075-1 | phenylethyl acetate biosynthesis | 1 |
| Total |  | 108 |

| **Pathway ID** | **Specific pathway at stage 5** | **No. of genes** |
| --- | --- | --- |
| BGALACT-PWY-60 | Lactose degradation III | 19 |
| PWY-181 | Photorespiration | 13 |
| PWY-6842-12 | Glutathione-mediated detoxification II | 12 |
| PWY-5464 | Superpathway of cytosolic glycolysis (plants), pyruvate dehydrogenase and TCA cycle | 10 |
| PWY-6737-58 | Starch degradation V | 10 |
| PWY0-1319 | CDP-diacylglycerol biosynthesis II | 10 |
| PWY-2541 | Plant sterol biosynthesis | 9 |
| ETHYL-PWY-35 | Ethylene biosynthesis I (plants) | 8 |
| PWY-5910-3 | Superpathway of geranylgeranyldiphosphate biosynthesis I | 6 |
| GLUCONEO-PWY | Gluconeogenesis I | 6 |
| PWY-622 | Starch biosynthesis | 6 |
| PWY1F-FLAVSYN-39 | Flavonoid biosynthesis | 5 |
| PWY-6348 | Phosphate acquisition | 5 |
| PWY0-1335-16 | NADH to cytochrome bo oxidase electron transfer | 4 |
| PWY-6475-33 | Trans-lycopene biosynthesis II | 4 |
| FASYN-ELONG-PWY-26 | Fatty acid elongation -- saturated | 4 |
| PWY-6352 | 3-phosphoinositide biosynthesis | 4 |
| PWY-735-20 | Jasmonic acid biosynthesis | 4 |
| PWY-381 | Nitrate reduction II | 4 |
| ETOH-ACETYLCOA-ANA-PWY | Ethanol degradation I | 4 |
| GLYCOCAT-PWY | Glycogen degradation I | 4 |
| PWY-7238 | Sucrose biosynthesis II | 4 |
| PWY-5136 | Fatty acid &beta;-oxidation II | 4 |
| PWY-6803-25 | Phosphatidylcholine acyl editing | 3 |
| PWY-6900-10 | (Z)-butanethiol-S-oxide biosynthesis | 3 |
| PWY-5070-17 | Gibberellin biosynthesis I | 3 |
| PWY-1121 | Suberin monomers biosynthesis | 3 |
| PWY-6515 | Phloridzin biosynthesis | 3 |
| PWY-5787 | Oligomeric urushiol biosynthesis | 3 |
| PWY-1001 | Cellulose biosynthesis | 3 |
| PWY-1081-59 | Homogalacturonan degradation | 3 |
| PWY-6539 | Chorismate biosynthesis from 3-dehydroquinate | 3 |
| PWY-6323-35 | Benzoylanthranilate biosynthesis | 2 |
| PWY-6902-58 | Chitin degradation II | 2 |
| PWY-5349 | Esculetin biosynthesis | 2 |
| PWY-6527 | Stachyose degradation | 2 |
| PWY-7039 | Phosphatidate metabolism, as a signaling molecule | 2 |
| PWY1F-823 | Leucopelargonidin and leucocyanidin biosynthesis | 2 |
| PWY-6433 | Hydroxylated fatty acid biosynthesis | 2 |
| GLYCOGENSYNTH-PWY | Glycogen biosynthesis I | 2 |
| GLYSYN-PWY | Glycine biosynthesis I | 2 |
| PWY-7219 | Adenosine ribonucleotides de novo biosynthesis | 2 |
| PWY-5142 | Acyl-ACP thioesterase pathway | 2 |
| PWY-6901 | Glucose and xylose degradation | 2 |
| PWY-6539 | Petivericin biosynthesis | 2 |
| PWY-7214-8 | Baicalein degradation | 2 |
| PWY-5046 | Pyruvate decarboxylation to acetyl CoA | 2 |
| TRIGLSYN-PWY | Triacylglycerol biosynthesis | 1 |
| PWY-241 | C4 photosynthetic carbon assimilation cycle | 1 |
| PWY-5148 | Acyl-CoA hydrolysis | 1 |
| PWY-5461-56 | 2-oxoisovalerate decarboxylation to isobutanoyl-CoA | 1 |
| COA-PWY-109 | Betanidin degradation | 1 |
| PWY-5176-90 | Coenzyme A biosynthesis | 1 |
| CYANCAT-PWY-38 | Coumarin biosynthesis (via 2-coumarate) | 1 |
| GLUCONSUPER-PWY-146 | Cyanate degradation | 1 |
| PWY-5859-37 | D-gluconate degradation | 1 |
| PWY-6692-14 | Eugenol and isoeugenol biosynthesis | 1 |
| PWY-3821 | Fe(II) oxidation | 1 |
| PWY-5829 | Galactose degradation III | 1 |
| PWY-4321-54 | Geraniol and geranial biosynthesis | 1 |
| PWY-6606-162 | Glutamate degradation IV | 1 |
| LCYSDEG-PWY-56 | Guanosine nucleotides degradation II | 1 |
| LEUSYN-PWY-49 | L-cysteine degradation II | 1 |
| PWY-5076-142 | Leucine biosynthesis | 1 |
| PWY-5466-24 | Leucine degradation III | 1 |
| PWY-702-128 | Matairesinol biosynthesis | 1 |
| PWY-6303-1 | Methionine biosynthesis II | 1 |
| NONMEVIPP-PWY | Mmethyl indole-3-acetate interconversion | 1 |
| PWY-922-59 | Methylerythritol phosphate pathway | 1 |
| PWY-5653-107 | Mevalonate pathway I | 1 |
| PHESYN-130 | NAD biosynthesis from 2-amino-3- semialdehyde | 1 |
| LIPASYN-PWY-33 | Phenylalanine biosynthesis I | 1 |
| PWY-5059 | Phospholipases | 1 |
| PWY-6710-64 | Pinobanksin biosynthesis | 1 |
| PWY-7262-28 | Poly-hydroxy fatty acids biosynthesis | 1 |
| PWY-6151-29 | Rose anthocyanin biosynthesis II (via cyanidin 3-O-&beta;-D-glucoside) | 1 |
| BSUBPOLYAMSYN-PWY-50 | S-adenosyl-L-methionine cycle I | 1 |
| PWY-5464-3 | Spermidine biosynthesis I | 1 |
| PWY-7228-20 | Superpathway of cytosolic glycolysis (plants), pyruvate dehydrogenase and TCA cycle | 1 |
| SER-GLYSYN-PWY | Superpathway of guanosine nucleotides de novo biosynthesis I | 1 |
| PWY0-1466-61 | Superpathway of serine and glycine biosynthesis I | 1 |
| LIPAS-PWY | Trehalose degradation VI (periplasmic) | 1 |
| TRPSYN-PWY-48 | Triacylglycerol degradation | 1 |
| VALDEG-PWY-145 | Tryptophan biosynthesis | 1 |
| PWY-5944-93 | Valine degradation I | 1 |
| PWY-5944-93 | Zeaxanthin biosynthesis | 1 |
| PWY-6351 | D-myo-inositol (1,4,5)-trisphosphate biosynthesis | 1 |
| PWY-6950 | DIMBOA-glucoside biosynthesis | 1 |
| PWY0-1544-23 | Proline to cytochrome bo oxidase electron transfer | 1 |
| PWY0-541-31 | cyclopropane fatty acid (CFA) biosynthesis | 1 |
| PWY-7250-38 | [2Fe-2S] iron-sulfur cluster biosynthesis | 1 |
| PWY-7170-39 | phytochromobilin biosynthesis | 1 |
| Total |  | 261 |
