# Supplementary material for: Transcriptome Profiling of Petal Abscission Zone and Functional Analysis of an Aux/IAA Family Gene RhIAA16 Involved in Petal Shedding in Rose
Source: Front Plant Sci. 2016 Sep 15;7:1375. doi: 10.3389/fpls.2016.01375 (PMC5023668; doi:10.3389/fpls.2016.01375)
Supplement: TABLE S5 — Differentially transcribed transcription factor. [file Table_5.DOCX]

***Supplementary Material***

**Transcriptome profiling of petal abscission zone and functional analysis of an Aux/IAA family gene *RhIAA16* involved in petal shedding in rose**

**Yuerong Gao, Chun Liu, Xiaodong Li, Haiqian Xu, Yue Liang, Nan Ma, Zhangjun Fei, Junping Gao, Cai-Zhong Jiang, Chao Ma**

***Correspondence:**

Chao Ma ([mac@cau.edu.cn](mailto:mac@cau.edu.cn)) & Cai-Zhong Jiang ([cjiang@ucdavis.edu](mailto:cjiang@ucdavis.edu))

**Supplementary Table 5 Differentially transcribed transcription factors**

| **GeneID** | **annotation** | **Stage 3** | **Stage 5** | **Ratio Stage5/3** | **Adjust p** |
| --- | --- | --- | --- | --- | --- |
| **Zinc finger** | | | | | |
| RSA05546 | Zinc finger CCCH domain-containing protein | 22.78 | 10.62 | 0.47 | 3.63E-05 |
| RSA06944 | Zinc finger protein CONSTANS-LIKE 1 | 31.65 | 15.96 | 0.5 | 0.000547484 |
| RSA07010 | Zinc finger protein CONSTANS-like protein | 8.31 | 44.71 | 5.38 | 1.13E-14 |
| RSA07011 | Zinc finger protein CONSTANS-like protein | 9.66 | 49.71 | 5.14 | 1.51E-14 |
| RSA07767 | Zinc finger protein | 34.29 | 70.53 | 2.06 | 0.001167204 |
| RSA07768 | Zinc finger protein | 25.67 | 52.73 | 2.05 | 0.012476026 |
| RSA08171 | Zinc finger CCCH domain-containing protein 29-like protein | 28.23 | 60.74 | 2.15 | 0.000471431 |
| RSA08227 | Dof zinc finger protein | 32.04 | 65.3 | 2.04 | 0.004243998 |
| RSA08228 | Dof zinc finger protein | 31.06 | 64.55 | 2.08 | 0.003141572 |
| RSA10082 | C2H2 zinc finger protein | 8.97 | 2.59 | 0.29 | 0.005628554 |
| RSA13075 | Zinc finger protein | 9.14 | 0.89 | 0.1 | 2.79E-05 |
| RSA21908 | Zinc finger protein | 1.51 | 40.06 | 26.53 | 2.62E-15 |
| RSA25659 | Zinc finger protein LSD1 | 6.37 | 27.2 | 4.27 | 0.000587635 |
| RSA29201 | C2H2 zinc finger protein | 1.39 | 8.98 | 6.45 | 0.032390461 |
| RSA34390 | Dof zinc finger protein | 29.09 | 3.78 | 0.13 | 0.000932146 |
| RSA37492 | Zinc finger homeodomain protein 1 | 555.25 | 193.74 | 0.35 | 7.20E-13 |
| RSA46156 | Dof zinc finger protein 4 | 9.04 | 35.14 | 3.89 | 1.24E-05 |
| RSA49485 | RING zinc finger family protein | 8.63 | 1.81 | 0.21 | 0.018354261 |
| RSA51768 | CONSTANS-like zinc finger protein | 13.93 | 27.95 | 2.01 | 0.014492993 |
| RSA51769 | CONSTANS-like zinc finger protein | 14.39 | 29.42 | 2.04 | 0.010041232 |
| RSA56647 | Zinc finger protein CONSTANS-LIKE 1 | 32.74 | 16.26 | 0.5 | 0.000378131 |
| RSA56947 | Zinc finger protein CONSTANS-like protein | 9.47 | 58.4 | 6.17 | 6.35E-13 |
| RSA59690 | Zinc finger protein | 35.62 | 75.09 | 2.11 | 0.000777905 |
| **WRKY family** | | | | | |
| RSA03365 | WRKY transcription factor, putative | 7.68 | 29.21 | 3.8 | 0.003792875 |
| RSA03897 | WRKY transcription factor 1 | 8.82 | 28.45 | 3.23 | 5.17E-05 |
| RSA05615 | WRKY transcription factor, putative | 5.72 | 12.6 | 2.2 | 0.027154471 |
| RSA05617 | WRKY transcription factor, putative | 5.8 | 13.4 | 2.31 | 0.012094834 |
| RSA09969 | WRKY transcription factor | 27.49 | 80.6 | 2.93 | 0.0033098 |
| RSA44295 | WRKY transcription factor | 33.59 | 103.28 | 3.07 | 7.99E-09 |
| RSA44296 | WRKY transcription factor | 40.17 | 116.85 | 2.91 | 3.37E-08 |
| RSA44297 | WRKY transcription factor 26 | 18.59 | 40.11 | 2.16 | 0.012111486 |
| RSA53833 | WRKY transcription factor 31 | 5.45 | 16.52 | 3.03 | 0.000112694 |
| RSA53834 | WRKY transcription factor 23 | 6.35 | 18.61 | 2.93 | 0.002207349 |
| RSA53836 | WRKY transcription factor 31 | 4.02 | 15.01 | 3.74 | 0.006433249 |
| RSA54472 | WRKY transcription factor, putative | 5.38 | 22.13 | 4.11 | 5.44E-08 |
| RSA54473 | WRKY transcription factor 1 | 6.14 | 25.74 | 4.19 | 8.57E-09 |
| RSA59196 | WRKY transcription factor 08 | 13.61 | 49.17 | 3.61 | 0.020525731 |
| RSA59197 | WRKY transcription factor 08 | 14.33 | 50.15 | 3.5 | 0.022678177 |
| RSA59198 | WRKY family transcription factor family protein | 13.96 | 49.78 | 3.57 | 0.019990304 |
| RSA59199 | WRKY family transcription factor family protein | 15.48 | 53.33 | 3.45 | 0.026491227 |
| RSA68622 | WRKY transcription factor 6 family protein | 13.54 | 46.31 | 3.42 | 5.96E-07 |
| RSA68623 | WRKY transcription factor 31 | 24.35 | 84.92 | 3.49 | 5.58E-06 |
| RSA72683 | WRKY transcription factor 45 | 0 | 8.1 | 809.67 | 1.32E-05 |
| **ERF** | | | | | |
| RSA28901 | Ethylene responsive transcription factor 2a | 4.08 | 24.01 | 5.88 | 5.76E-06 |
| RSA33267 | Ethylene-responsive transcription factor 7 | 23.8 | 91.96 | 3.86 | 3.60E-08 |
| RSA37531 | Ethylene-responsive transcription factor | 16.17 | 62.1 | 3.84 | 4.66E-11 |
| RSA38284 | Ethylene-responsive transcription factor | 64.4 | 16.98 | 0.26 | 8.97E-11 |
| RSA41341 | Ethylene-responsive transcription factor 6 | 154.45 | 424.3 | 2.75 | 0.000280204 |
| RSA41868 | Ethylene-responsive transcription factor 1 | 8.61 | 32.75 | 3.8 | 0.0001797 |
| RSA42290 | Ethylene-responsive transcription factor 12 | 26.57 | 3.79 | 0.14 | 7.94E-12 |
| RSA44839 | Ethylene responsive transcription factor | 5.89 | 36.98 | 6.28 | 0.001313539 |
| RSA47048 | Ethylene-responsive transcription factor | 19.2 | 115.54 | 6.02 | 0.0009047 |
| RSA47049 | Ethylene-responsive transcription factor | 20.02 | 120.49 | 6.02 | 0.0007967 |
| RSA47050 | Ethylene-responsive transcription factor | 18.91 | 113.78 | 6.02 | 0.0009334 |
| RSA47051 | Ethylene-responsive transcription factor | 21.18 | 121.91 | 5.76 | 0.0008476 |
| RSA47052 | Ethylene-responsive transcription factor | 25.18 | 149.25 | 5.93 | 0.0014577 |
| RSA49687 | Ethylene responsive transcription factor 2b | 3.93 | 10.15 | 2.58 | 0.040221576 |
| RSA49689 | Ethylene responsive transcription factor 2b | 4.38 | 11.31 | 2.58 | 0.036572461 |
| RSA50970 | Ethylene-responsive transcription factor 7 | 5.21 | 58.45 | 11.22 | 0.0001211 |
| RSA54041 | Ethylene-responsive transcription factor 5 | 21.28 | 58.1 | 2.73 | 0.000698971 |
| RSA54042 | Ethylene-responsive transcription factor 3 | 24.69 | 74.96 | 3.04 | 0.002269416 |
| RSA49076 | ERF/AP2 transcription factor family | 91.68 | 36.56 | 0.4 | 3.35E-07 |
| **Aux/IAA** | | | | | |
| RSA04500 | Aux/IAA27 | 33.19 | 88.58 | 2.67 | 8.37E-07 |
| RSA05184 | Aux/IAA1-like | 5.96 | 0.67 | 0.11 | 0.011598385 |
| RSA33069 | Aux/IAA16 | 80.94 | 165.03 | 2.04 | 0.015565688 |
| RSA37816 | Aux/IAA13 isoform X1 | 13.14 | 6.55 | 0.5 | 0.00328157 |
| RSA37817 | Aux/IAA13 isoform X1 | 13.3 | 6.68 | 0.5 | 0.00353295 |
| RSA38191 | Aux/IAA27-like | 28.52 | 101.47 | 3.56 | 2.57E-07 |
| RSA45026 | Aux/IAA28-like | 394.68 | 168.36 | 0.43 | 1.56E-08 |
| RSA45027 | Aux/IAA28-like | 411.14 | 173.63 | 0.42 | 9.78E-09 |
| RSA45028 | Aux/IAA28-like | 394.48 | 169.38 | 0.43 | 2.00E-08 |
| RSA45029 | Aux/IAA28-like | 416.25 | 177.08 | 0.43 | 1.25E-08 |
| RSA45030 | Aux/IAA27-like | 20.59 | 81.81 | 3.97 | 5.51E-05 |
| RSA47413 | Aux/IAA26-like | 5.37 | 19.73 | 3.67 | 8.49E-06 |
| RSA52969 | Aux/IAA8-like | 62.56 | 27.42 | 0.44 | 0.000674497 |
| RSA63979 | Aux/IAA13 isoform X1 | 7.96 | 16.83 | 2.11 | 0.049311724 |
| **bHLH** | | | | | |
| RSA18230 | Transcription factor bHLH | 0.3 | 5.52 | 18.21 | 0.039238434 |
| RSA34443 | Transcription factor bHLH | 23.49 | 50.97 | 2.17 | 0.010742446 |
| RSA37014 | Transcription factor bHLH | 30.03 | 140.1 | 4.67 | 2.42E-13 |
| RSA49335 | Transcription factor bHLH96 | 65.32 | 31.43 | 0.48 | 6.56E-05 |
| RSA49336 | Transcription factor bHLH96 | 58.76 | 27.88 | 0.47 | 2.69E-05 |
| RSA49337 | Transcription factor bHLH96 | 54.16 | 25.62 | 0.47 | 4.07E-05 |
| RSA51607 | Transcription factor bHLH30 | 9.45 | 19.44 | 2.06 | 0.031709593 |
| RSA59554 | Transcription factor bHLH | 267.75 | 92.48 | 0.35 | 1.65E-12 |
| RSA59555 | Transcription factor bHLH | 251.65 | 86.46 | 0.34 | 1.33E-12 |
| RSA59558 | Transcription factor bHLH | 206.32 | 72.36 | 0.35 | 3.07E-12 |
| RSA59559 | Transcription factor bHLH | 193.68 | 67.65 | 0.35 | 2.64E-12 |
| RSA07989 | Transcription factor bHLH | 22.64 | 10.49 | 0.46 | 0.00309227 |
| **Trihelix** | | | | | |
| RSA37338 | Trihelix transcription factor GT-2 | 21.33 | 8.95 | 0.42 | 0.007725806 |
| RSA39525 | trihelix transcription factor ASIL2 | 8.74 | 3.23 | 0.37 | 0.041026996 |
| RSA69269 | Trihelix transcription factor GT-2 | 130.78 | 56.81 | 0.43 | 3.17E-08 |
| RSA69270 | Trihelix transcription factor GT-2 | 97.95 | 41.54 | 0.42 | 7.98E-08 |
| RSA69271 | Trihelix transcription factor GT-2 | 124.72 | 53.65 | 0.43 | 1.37E-08 |
| RSA69272 | Trihelix transcription factor GT-2 | 128.81 | 56.37 | 0.44 | 2.62E-08 |
| RSA69273 | Trihelix transcription factor GT-2 | 127.79 | 54.6 | 0.43 | 1.82E-08 |
| RSA69274 | Trihelix transcription factor GT-2 | 127.76 | 55.18 | 0.43 | 2.66E-08 |
| RSA69275 | Trihelix transcription factor GT-2 | 127.35 | 56.76 | 0.45 | 9.47E-08 |
| RSA69276 | Trihelix transcription factor GT-2 | 124.39 | 53.97 | 0.43 | 3.79E-08 |
| RSA69277 | Trihelix transcription factor GT-2 | 127.28 | 54.26 | 0.43 | 1.30E-08 |
| RSA69278 | Trihelix transcription factor GT-2 | 124 | 54.96 | 0.44 | 8.15E-08 |
| **HB** | | | | | |
| RSA08222 | Homeobox protein knotted-1-like 3 | 11.72 | 25.85 | 2.21 | 0.007805075 |
| RSA25553 | homeobox protein 26 (HB26); | 4.69 | 24.78 | 5.29 | 4.83E-05 |
| RSA47028 | Homeobox-leucine zipper-like protein | 95.65 | 215.52 | 2.25 | 4.77E-05 |
| RSA47130 | Homeobox-leucine zipper family protein | 383.62 | 130.03 | 0.34 | 3.47E-13 |
| RSA48341 | Homeobox-leucine zipper-like protein | 11.38 | 38.13 | 3.35 | 1.04E-06 |
| RSA66390 | Homeobox-leucine zipper protein ANTHOCYANINLESS 2 | 38.2 | 80.32 | 2.1 | 0.000658519 |
| RSA66391 | Homeobox-leucine zipper family protein | 38.36 | 80.63 | 2.1 | 0.000663604 |
| RSA77904 | Homeobox-leucine zipper-like protein | 51.99 | 7.89 | 0.15 | 1.81E-14 |
| RSA77920 | Homeobox-leucine zipper-like protein | 157.03 | 22.55 | 0.14 | 1.28E-28 |
| **BTB/POZ** | | | | | |
| RSA30901 | BTB/POZ domain-containing protein | 8.04 | 20.42 | 2.54 | 0.007691675 |
| RSA39178 | BTB/POZ domain-containing protein | 14.78 | 54.62 | 3.7 | 1.50E-09 |
| RSA40968 | BTB/POZ domain-containing protein | 31.02 | 90.22 | 2.91 | 2.82E-08 |
| RSA47203 | BTB/POZ and TAZ domain-containing protein 2 | 22.64 | 9.06 | 0.4 | 0.002630852 |
| RSA56101 | BTB/POZ domain-containing protein | 36.68 | 16.81 | 0.46 | 1.06E-05 |
| RSA56102 | BTB/POZ domain-containing protein | 27.59 | 12.75 | 0.46 | 3.68E-05 |
| RSA56180 | BTB/POZ domain-containing protein | 26.78 | 9.49 | 0.35 | 1.46E-07 |
| RSA61690 | BTB/POZ domain-containing protein | 14.18 | 5.72 | 0.4 | 0.000129374 |
| **MYB** | | | | | |
| RSA26674 | MYB transcription factor | 131.77 | 22.94 | 0.17 | 1.38E-10 |
| RSA29532 | MYB transcription factor | 5.57 | 18.56 | 3.33 | 0.002823907 |
| RSA31373 | MYB transcription factor | 143.16 | 11.12 | 0.08 | 5.95E-43 |
| RSA31374 | MYB transcription factor | 156.05 | 11.78 | 0.08 | 1.34E-44 |
| RSA39324 | MYB transcription factor R2R3-like protein | 4.14 | 25.45 | 6.15 | 3.07E-10 |
| RSA45917 | MYB transcription factor | 894.65 | 292.72 | 0.33 | 4.23E-10 |
| RSA50533 | Myb family transcription factor | 12.79 | 29.65 | 2.32 | 0.003971928 |
| RSA50534 | Myb family transcription factor | 11.24 | 24.97 | 2.22 | 0.018682945 |
| **BZIP** | | | | | |
| RSA11925 | BZIP transcription factor family protein | 20.57 | 8.84 | 0.43 | 0.008217812 |
| RSA37867 | BZIP transcription factor family protein | 14.19 | 4.74 | 0.33 | 9.43E-05 |
| RSA63791 | BZIP transcription factor family protein | 38.67 | 1.03 | 0.03 | 2.30E-41 |
| RSA63792 | BZIP transcription factor family protein | 36.72 | 0.98 | 0.03 | 1.73E-42 |
| RSA63793 | BZIP transcription factor family protein | 40.87 | 1.11 | 0.03 | 1.00E-42 |
| RSA63794 | BZIP transcription factor family protein | 57.62 | 2.27 | 0.04 | 1.21E-30 |
| RSA63795 | BZIP transcription factor family protein | 35.98 | 1 | 0.03 | 3.70E-42 |
| **NAC** | | | | | |
| RSA05211 | NAC domain protein | 24.79 | 52.7 | 2.13 | 0.00757673 |
| RSA05995 | NAC domain protein, | 11.91 | 5.58 | 0.47 | 0.033236148 |
| RSA12529 | NAC domain protein | 18.03 | 40.95 | 2.27 | 0.023737306 |
| RSA35239 | NAC domain protein | 0 | 4.07 | 407.33 | 0.042554091 |
| RSA40770 | NAC transcription factor 097 | 7.17 | 18.98 | 2.65 | 0.023574252 |
| RSA45753 | NAC domain protein | 52.23 | 112.48 | 2.15 | 0.000732562 |
| **SCL** | | | | | |
| RSA27469 | Scarecrow-like transcription factor 3 family protein | 4.91 | 15.53 | 3.16 | 5.46E-05 |
| RSA27470 | Scarecrow-like transcription factor 3 family protein | 4.79 | 15.01 | 3.14 | 6.21E-05 |
| RSA27471 | Scarecrow-like transcription factor 3 family protein | 4.94 | 15.58 | 3.15 | 5.11E-05 |
| **GATA** | | | | | |
| RSA34812 | GATA transcription factor | 85.39 | 9.01 | 0.11 | 1.91E-07 |
| RSA34813 | GATA transcription factor | 82.05 | 8.68 | 0.11 | 2.68E-07 |
| RSA54848 | GATA transcription factor | 75.24 | 8.5 | 0.11 | 2.07E-09 |
| **HSF** | | | | | |
| RSA08672 | Heat Stress Transcription Factor family protein | 21.1 | 5.25 | 0.25 | 3.36E-11 |
| RSA63882 | Heat Stress Transcription Factor family protein | 18.29 | 4.91 | 0.27 | 7.61E-10 |
| RSA63883 | Heat Stress Transcription Factor family protein | 20.35 | 7.68 | 0.38 | 0.021623629 |
| **ARF** | | | | | |
| RSA47053 | Auxin response factor 3 family protein | 35.9 | 14.91 | 2.41 | 9.70E-04 |
| **PLATZ** | | | | | |
| RSA00994 | PLATZ transcription factor family protein | 8.78 | 1.21 | 0.14 | 0.011197901 |
| **MADS** | | | | | |
| RSA79623 | Transcription factor MADS box | 5.19 | 0.7 | 0.14 | 0.002837259 |
