# Supplementary material for: Transcriptome Profiling of Petal Abscission Zone and Functional Analysis of an Aux/IAA Family Gene RhIAA16 Involved in Petal Shedding in Rose
Source: Front Plant Sci. 2016 Sep 15;7:1375. doi: 10.3389/fpls.2016.01375 (PMC5023668; doi:10.3389/fpls.2016.01375)
Supplement: TABLE S6 — Differentially transcribed genes related to hormones. [file Table_6.DOCX]

***Supplementary Material***

**Transcriptome profiling of petal abscission zone and functional analysis of an Aux/IAA family gene *RhIAA16* involved in petal shedding in rose**

**Yuerong Gao, Chun Liu, Xiaodong Li, Haiqian Xu, Yue Liang, Nan Ma, Zhangjun Fei, Junping Gao, Cai-Zhong Jiang, Chao Ma**

***Correspondence:**

Chao Ma ([mac@cau.edu.cn](mailto:mac@cau.edu.cn)) & Cai-Zhong Jiang ([cjiang@ucdavis.edu](mailto:cjiang@ucdavis.edu))

**Supplementary Table 6 Differentially transcribed genes related to hormones**

| **GeneID** | **Annotation** | | **Stage 3** | **Stage 5** | **Ratio Stage 5/3** | **Adjust p** |
| --- | --- | --- | --- | --- | --- | --- |
| **Genes related to ethylene pathway** | | | | | | |
| **ACC oxidase** | | |  |  |  |  |
| RSA49300 | | 1-aminocyclopropane-1-carboxylate oxidase | 77.66 | 244.71 | 3.15 | 3.56E-10 |
| RSA49301 | | 1-aminocyclopropane-1-carboxylate oxidase | 99.42 | 298.16 | 3 | 1.45E-09 |
| RSA49302 | | 1-aminocyclopropane-1-carboxylate oxidase | 136.64 | 384.61 | 2.81 | 1.00E-08 |
| RSA49299 | | 1-aminocyclopropane-1-carboxylate oxidase | 159.31 | 441.53 | 2.77 | 1.46E-08 |
| RSA65671 | | 1-aminocyclopropane-1-carboxylate oxidase 4 | 6.22 | 17.09 | 2.75 | 0.0022522 |
| RSA65670 | | 1-aminocyclopropane-1-carboxylate oxidase 4 | 8.4 | 22.48 | 2.68 | 0.0118225 |
| RSA48324 | | 1-aminocyclopropane-1-carboxylate oxidase homolog | 12.84 | 33.55 | 2.61 | 0.0020963 |
| RSA65667 | | 1-aminocyclopropane-1-carboxylate oxidase 4 | 7.17 | 18.48 | 2.58 | 0.0042085 |
| RSA46967 | | 1-aminocyclopropane-1-carboxylate oxidase-1-like protein | 5.66 | 14.47 | 2.56 | 0.0134638 |
| RSA46966 | | 1-aminocyclopropane-1-carboxylate oxidase-1-like protein | 8.86 | 22.45 | 2.53 | 0.0222229 |
| RSA57927 | | 1-aminocyclopropane-1-carboxylate deaminase | 21.8 | 11.59 | 0.53 | 0.0015861 |
| RSA07669 | | 1-aminocyclopropane-1-carboxylate oxidase | 77.64 | 33.95 | 0.44 | 0.0003405 |
| RSA59483 | | 1-aminocyclopropane-1-carboxylate oxidase | 237.08 | 90.62 | 0.38 | 0.0023509 |
| RSA45707 | | 1-aminocyclopropane-1-carboxylate oxidase | 286.56 | 91.68 | 0.32 | 0.0029605 |
| RSA19163 | | 1-aminocyclopropane-1-carboxylate oxidase-like protein 1 | 18.55 | 5.15 | 0.28 | 0.0202525 |
| **Ethylene-responsive transcription factor** | | | | | | |
|  |  | |  |  |  |  |
| RSA28901 | Ethylene responsive transcription factor 2a | | 4.08 | 24.01 | 5.88 | 5.76E-06 |
| RSA33267 | Ethylene-responsive transcription factor 7 | | 23.8 | 91.96 | 3.86 | 3.60E-08 |
| RSA37531 | Ethylene-responsive transcription factor | | 16.17 | 62.1 | 3.84 | 4.66E-11 |
| RSA38284 | Ethylene-responsive transcription factor | | 64.4 | 16.98 | 0.26 | 8.97E-11 |
| RSA41341 | Ethylene-responsive transcription factor 6 | | 154.45 | 424.3 | 2.75 | 0.000280204 |
| RSA41868 | Ethylene-responsive transcription factor 1 | | 8.61 | 32.75 | 3.8 | 0.0001797 |
| RSA42290 | Ethylene-responsive transcription factor 12 | | 26.57 | 3.79 | 0.14 | 7.94E-12 |
| RSA44839 | Ethylene responsive transcription factor | | 5.89 | 36.98 | 6.28 | 0.001313539 |
| RSA47048 | Ethylene-responsive transcription factor | | 19.2 | 115.54 | 6.02 | 0.000904697 |
| RSA47049 | Ethylene-responsive transcription factor | | 20.02 | 120.49 | 6.02 | 0.000796729 |
| RSA47050 | Ethylene-responsive transcription factor | | 18.91 | 113.78 | 6.02 | 0.000933431 |
| RSA47051 | Ethylene-responsive transcription factor | | 21.18 | 121.91 | 5.76 | 0.000847647 |
| RSA47052 | Ethylene-responsive transcription factor | | 25.18 | 149.25 | 5.93 | 0.001457674 |
| RSA49687 | Ethylene responsive transcription factor 2b | | 3.93 | 10.15 | 2.58 | 0.040221576 |
| RSA49689 | Ethylene responsive transcription factor 2b | | 4.38 | 11.31 | 2.58 | 0.036572461 |
| RSA50970 | Ethylene-responsive transcription factor 7 | | 5.21 | 58.45 | 11.22 | 0.000121081 |
| RSA54041 | Ethylene-responsive transcription factor 5 | | 21.28 | 58.1 | 2.73 | 0.000698971 |
| RSA54042 | Ethylene-responsive transcription factor 3 | | 24.69 | 74.96 | 3.04 | 0.002269416 |
| RSA49076 | ERF/AP2 transcription factor family | | 91.68 | 36.56 | 0.4 | 3.35E-07 |
| **Ethylene receptor** | | | | | | |
| RSA06585 | Ethylene receptor | | 8.15 | 29.62 | 3.63 | 1.41E-05 |
| RSA35460 | Ethylene receptor 1 | | 9.97 | 30.08 | 3.02 | 0.0014723 |
| RSA35459 | Ethylene receptor 1 | | 8.69 | 25.67 | 2.96 | 0.0031993 |
| **SAM synthetase** | | | | | | |
| RSA41418 | S-adenosylmethionine synthase | | 1452.46 | 899.34 | 0.62 | 0.0280673 |
| **Genes related to ABA pathway** | | |  |  |  |  |
| RSA38462 | Abscisic acid receptor PYL4 | | 52.88 | 32.27 | 0.61 | 0.0406811 |
| **Genes related to cytokinin pathway** | | |  |  |  |  |
| RSA31502 | Cytokinin riboside 5'-monophosphate phosphoribohydrolase-like | | 17.93 | 33.79 | 1.89 | 0.0414261 |
| **Genes related to gibberellin pathway** | | |  |  |  |  |
| **Gibberellin 2-oxidase** | | |  |  |  |  |
| RSA43716 | Gibberellin 2-oxidase | | 32.69 | 121.42 | 3.71 | 1.91E-05 |
| RSA43715 | Gibberellin 2-oxidase | | 28.38 | 102.5 | 3.61 | 2.03E-05 |
| **Gibberellin receptor** | | |  |  |  |  |
| RSA43504 | Gibberellin receptor GID1, putative | | 100.72 | 556.1 | 5.52 | 4.23E-24 |
| RSA39340 | Gibberellin receptor GID1, putative | | 3.88 | 10.31 | 2.66 | 0.037676 |
| RSA40786 | Gibberellin receptor GID1, putative | | 267.43 | 121.9 | 0.46 | 1.35E-07 |
| **Gibberellin-regulated protein** | | |  |  |  |  |
| RSA37317 | Gibberellin-regulated protein | | 660.89 | 5082.52 | 7.69 | 4.81E-20 |
| RSA60435 | Gibberellin-regulated protein | | 16.91 | 6.64 | 0.39 | 0.0355939 |
| RSA60433 | Gibberellin-regulated protein | | 16.64 | 6.64 | 0.4 | 0.0409723 |
| RSA38230 | Gibberellin-regulated protein 6-like | | 7256.63 | 932.33 | 0.13 | 1.91E-16 |
| RSA07956 | Gibberellin-regulated protein | | 38.59 | 17.32 | 0.45 | 0.023147 |
| RSA07955 | Gibberellin-regulated protein | | 38.53 | 17.55 | 0.46 | 0.0237102 |
| RSA77909 | Gibberellin-regulated protein | | 5.49 | 192.65 | 35.09 | 3.23E-08 |
| **Genes related to jasmonic acid pathway** | | |  |  |  |  |
| RSA71412 | Jasmonate-induced protein | | 0.83 | 11.54 | 13.85 | 0.0005736 |
| RSA61121 | Jasmonate ZIM-domain protein 10 | | 36.84 | 97.71 | 2.65 | 4.52E-06 |
| RSA08103 | Jasmonate ZIM-domain protein 10 | | 34.41 | 89.71 | 2.61 | 9.29E-06 |
| RSA61124 | Jasmonate ZIM-domain protein 10 | | 38.25 | 93.25 | 2.44 | 0.0001508 |
| RSA34095 | Jasmonate ZIM domain protein j | | 104.06 | 24.98 | 0.24 | 0.00129 |
| RSA34094 | Jasmonate ZIM domain protein j | | 113.11 | 25.72 | 0.23 | 3.46E-06 |
